# Instrumentation Removal following Minimally Invasive Posterior Percutaneous Pedicle Screw-Rod Stabilization (PercStab) of Thoracolumbar Fractures Is Not Always Required

**DOI:** 10.1155/2020/7949216

**Published:** 2020-07-31

**Authors:** Neil Manson, Dana El-Mughayyar, Erin Bigney, Eden Richardson, Edward Abraham

**Affiliations:** ^1^Canada East Spine Centre, Saint John Regional Hospital, 400 University Ave, PO Box 2100, Saint John, New Brunswick E2L 4L4, Canada; ^2^Saint John Regional Hospital, Horizon Health Network, 400 University Ave, PO Box 2100, Saint John, New Brunswick E2L 4L4, Canada; ^3^Department of Surgery, Dalhousie University, 100 Tucker Park Rd, Saint John, New Brunswick E2K 5E2, Canada

## Abstract

**Background:**

Percutaneous stabilization for spinal trauma confers less blood loss, reduces postoperative pain, and is less invasive than open stabilization and fusion. The current standard of care includes instrumentation removal.

**Objective:**

1. Reporting patient outcomes following minimally invasive posterior percutaneous pedicle screw-rod stabilization (PercStab). 2. Evaluating the results of instrumentation retention.

**Methods:**

A prospective observational study of 32 consecutive patients receiving PercStab without direct decompression or fusion. Baseline data demographics were collected. Operative outcomes of interest were operative room (OR) time, blood loss, and length of hospital stay. Follow-up variables of interest included patient satisfaction, Numeric Rating Scales for Back and Leg (NRS-B/L) pain, Oswestry Disability Index (ODI), and return to work. Clinical outcome data (ODI and NRS-B/L) were collected at 3, 12, 24 months and continued at a 24-month interval up to a maximum of 8 years postoperatively.

**Results:**

81.25% of patients (*n* = 26) retained their instrumentation and reported minimal disability, mild pain, and satisfaction with their surgery and returned to work (mean = 6 months). Six patients required instrumentation removal due to prominence of the instrumentation or screw loosening, causing discomfort/pain. Instrumentation removal patients reported moderate back and leg pain until removal occurred; after removal, they reported minimal disability and mild pain. Neither instrumentation removal nor retention resulted in complications or further surgical intervention.

**Conclusions:**

PercStab without instrumentation removal provided high patient satisfaction, mild pain, and minimal disability and relieved the patient from the burden of finances and resources allocation of a second surgery.

## 1. Introduction

Spine fractures compromise approximately 6% of all fractures worldwide [1, 2]. Surgical treatments are proposed to patients with unstable traumatic pathologies, with or without neurological deficit [3]. While traditional open surgical treatments are commonly used, they may result in considerable blood loss, complications, extended hospital stays, and delayed functional recovery [4–7]. Minimally invasive surgical (MIS) techniques are intended to minimize approach morbidity and associated complications of open surgeries [5]. Posterior percutaneous pedicle screw fixation can provide spinal stabilization with placement through small incisions using specific MIS technologies, in an effort to minimize complications [8–11].

As this is a nonfusion technique, the current standard of care following minimally invasive posterior percutaneous pedicle screw-rod stabilization (PercStab) includes instrumentation removal [12]. There is no consensus on timing of hardware removal but it is considered once tissue healing occurs and stability is restored to prevent instrumentation failure, loosening at the bone-screw interface, or other instrument related complications [13, 14]. Vanek et al. showed that instrumentation was removed from the part of the lumbar spine (below L-2) between 12 and 18 months postoperatively [15, 16]. Yang et al. reported that seven patients of 64 opted out of having their instrumentation removed postoperatively as they reported feeling satisfied with their function and wished to avoid having a second procedure [11]. Satisfaction without any disruptions can eliminate a second procedure in patients.

The objectives of this study were (1) reporting patient outcomes following minimally invasive posterior percutaneous pedicle screw-rod stabilization to treat spine trauma and (2) evaluating the results of instrumentation retention.

## 2. Methods

A prospective observational study monitored 32 consecutive spine trauma patients meeting inclusion criteria: age of 18 years or older with unstable spinal trauma for which PercStab was the course of treatment. Baseline data collection included patient age, sex, body mass index (BMI), comorbidities, mechanism of injury (MOI), Injury Severity Score (ISS), AOSpine Classification system, numeric pain rating scale, and admission diagnosis. Operative variables of interest included operative room (OR) time, instrumentation type, blood loss, length of hospital stay, levels operated on and surgical morbidity/mortality. Outcome variables of interest are the Numeric Rating Scales for Back and Leg (NRS-B/L) pain, Oswestry Disability Index (ODI), patient satisfaction, and time to return to work. Clinical outcome data (ODI and NRS-B/L) were collected at 3, 12, 24 months and continued at a 24-month interval up to a maximum of 8 years postoperatively. Patient satisfaction was collected at the patient's final follow-up. When applicable, timing of instrumentation removal and reasoning was reported.

The AOSpine Classification system has been shown to have reasonable reliability and accuracy for clinical validation of a unified system to effectively communicate case-specific details of patient injuries [17, 18]. The ISS assesses severity of trauma and correlates with mortality and morbidity [19]. Patient satisfaction was measured through a 5-point Likert scale (1 = extremely dissatisfied, 2 = somewhat dissatisfied, 3 = neither satisfied nor dissatisfied, 4 = somewhat satisfied, and 5 = extremely satisfied) asking “Are you satisfied with the results of your surgery?”. The NRS-B/L and ODI questionnaire were used to quantify disability related to leg and back pain [20]. ODI has been shown to have excellent test-retest reliability [21]. Leg and back pain intensity (NRS-B/L) were measured through an 11-point numeric pain rating scale. Pain ratings represented the typical pain experienced over the preceding 24 hours, with potential scores ranging from 0 (“no pain”) to 10 (“worst pain imaginable”). Ratings are categorized as “mild” (0–3), “moderate” (4–6), or “severe” (7–10). The numeric pain rating scale has excellent test-retest reliability and responsiveness [22–24].

Instrumentation removal was not applied as a standard. Instrumentation removal was provided based on clinical complaint, physical exam findings, and imaging findings at the discretion of the surgeon and in shared decision-making with the patient. Without specific justification, instrumentation was not removed.

## 3. Surgical Technique

All patients received PercStab under general anesthesia. The patient was positioned prone on the OSI spine table with chest, hip, and leg bolsters placed to optimize alignment at the fracture site. Standard antiseptic skin preparation and draping was completed, and preoperative antibiotics were provided. Two C-arm fluoroscopy units provided simultaneous anteroposterior (AP) and lateral imaging capabilities. AP fluoroscopy guided placement of 1.5 cm stab incisions overlying each pedicle to be instrumented. The Pedicle Access Kit cannulated trochar (Medtronic Sofamor Danek) was tamped through the pedicle assuring that the tip of the trochar remained lateral to the medial pedicle wall at all times until the tip of the trochar passed into the vertebral body. Trochar position was confirmed on AP and lateral C-arm imaging. Guidewire was passed through the trochar followed by trochar removal. Guidewires were placed at each pedicle to be instrumented. The pedicles were then tapped, and cannulated screws were placed by hand.

The pedicle screw-rod construct was placed utilizing the Longitude or Sextant II systems (Medtronic Sofamor Danek; Legacy 5.5 titanium). The Sextant II system was utilized if the construct spanned one or two motion levels. The Longitude system was utilized if the construct was greater than two motion levels. Screws of 6.5 mm diameter were most commonly utilized. Screws were positioned to optimize construct stability and fracture alignment. If the spine and traumatic pathology permitted, screws were placed at the level of pathology as well. Bilateral titanium rods, 5.5 mm in diameter were measured, contoured, and passed in a subfascial plane through the pedicle screw extenders. Construct and fracture alignment were optimized with compression and distraction maneuvers, and final tightening of the set screws was completed. Insertion tools were removed. Final AP and lateral C-arm imaging were used to confirm the appropriate instrumentation placement and spinal alignment. Incisions were closed in layers and dressing was applied.

## 4. Results

The sample includes thirty-two consecutive trauma patients (24 males and 8 females; mean age 38.3 years; see Table 1 for demographics). The MOI for 75% of patients was motor vehicle crash (MVC) and the remaining 25% was caused by a fall. Of the sample, 40.62% had polytrauma with an ISS mean score of 9.5 showing moderate injury. Burst fractures account for 68.75% of the diagnoses; patient fracture diagnosis was classified using the AOSpine Classification System (Table 2). Within the sample, the presence of comorbidities was low with 15.62% of the sample impacted (see Table 1). Most patients received PercStab to treat spinal instability over a mean of 2 levels (range 1–6 levels).

PercStab as a surgical option resulted in no surgical induced morbidity/mortality but injury related morbidity preoperatively was seen in 21.87% of the sample which included superventricular tachycardia, blood abnormalities, urinary retention, low hemoglobin, sepsis, compromised respiratory function, and exacerbation of injuries due to poor compliance. Operative room morbidity was seen in 6.2% of the sample which included coagulation abnormalities and respiratory decline during intubation. Postoperative morbidity was seen in 35.9% of the sample which included poor pain control, hypervolemia, oxygen saturation fluctuations, low oxygen, *E. coli* in septum, staph epidemia in blood, fluid collection, ileus, staph aureus, and vomiting. The majority of patients were followed up for 48 months (time of final follow-up ranged from 4 to 8 years).

The majority of patients in this case series, 26 of 32 (19 males and 7 females; mean age of 40.7 years; see Table 1), did not require instrumentation to be removed; these patients will be referred to as the nonremoval group (NRG). Only 6 of 32 patients required instrumentation removal. The instrumentation removal group (IRG; 5 males and 1 female; mean age of 27.6 years; see Table 1) required removal due to radiologically confirmed screw loosening causing back pain or discomfort (4) or due to screw prominence causing discomfort with direct pressure (2). Time of removal ranged from 16 to 45 months. Patients who required removal completed a follow-up prior to their second surgery and were followed up for 24 months after removal. Operative details for both the NRG and IRG can be seen in Table 3.

**Figure 1:**
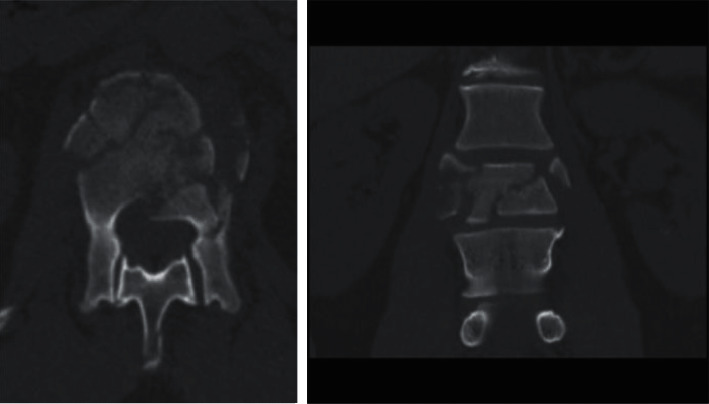
Axial and coronal CT scan of L1 burst fracture after a snowmobile accident.

**Figure 2:**
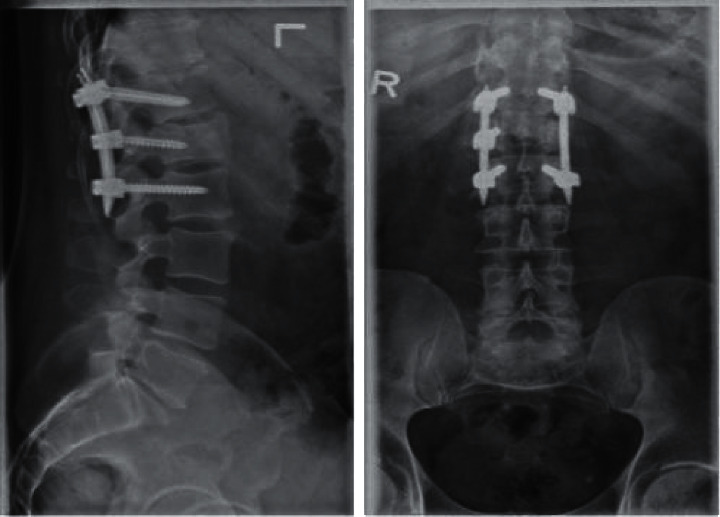
Lateral and AP X-ray of percutaneous stabilization of L1 burst fracture treated percutaneously at T12–L2 at 12-month follow-up.

Overall pain at baseline averaged 7 points. All patients show clinically meaningful reduction in pain from baseline to final follow-up from severe to mild. Patients who did not require removal on average reported moderate back and leg pain at 3 months postsurgery then mild back and leg pain at 12, 24, and 48 months (see Table 4). Patient presented with L1 burst fracture injury after a snowmobile accident (see Figure 1) is shown to have instrumentation retention at 12-month follow-up and back to rigorous activities (see Figure 2). Patients who required removal reported moderate back pain at all follow-up points until instrumental removal; after removal, reported back and leg pain dropped to mild (see Table 4). Reported ODI for both the NRG and IRG was within the lower range of moderate disability for 3, 12, and 24 months. At 48 months after surgery, the NRG reported minimal disability and 24 months after removal (48 months after surgery), the IRG reported minimal disability (see Table 4).

The median time to return to work following surgery reported by patients in the NRG was 6 months and 7 months for the IRG with all patients returning to full-time work by 12 months, aside from 5 patients who retired. All patients reported being somewhat satisfied to extremely satisfied with their surgery (NRG mean = 4.8, range = 4-5; IRG mean = 4.8, range = 4-5).

## 5. Discussion

Our case series supports the previous literature demonstrating favorable outcomes following MIS management of spinal trauma independent of removal or retention [11, 15, 25]. All patients showed positive outcomes following surgery, reporting minimal disability, mild pain, and high satisfaction with their surgery. PercStab is a reliable and accurate treatment for thoracic and lumbar spine fractures [26]. This case series demonstrated PercStab offers minimal surgical morbidity and blood loss similar to previous studies [27]. Minimally invasive percutaneous pedicle screw fixation without fusion for thoracolumbar fractures did not result in instrumentation failure or surgery-related complications [28] and resulted in patient satisfaction [29].

Previous research investigating PercStab as a treatment for thoracic and lumbar spine fractures followed a criteria of instrumentation removal as the standard of care; however, Yang et al. (2011) reported a small subset of their samples (10.94%) declined the second operation as the patients reported high satisfaction with their results [11]. The current case series was conducted to see how patients would fare if the standard was to retain the instrumentation, avoiding the patient burden, costs, and resource allocation that a second surgery requires. In the current case series, 81.25% of the sample retained their instrumentation and reported long-term minimal disability, mild pain, and satisfaction with their surgery.

Removal is usually done to avoid an instrumentation failure, loosening at the bone-screw interface, or other instrument related complications [25]. In the current case series, this occurred but in only 18.75% of the sample, suggesting that instrumentation removal could be decided based on clinical indications (loosening of screws, reported pain) instead of being the standard of treatment for all cases. In the current study, the primary indicator for instrumentation removal was patient-reported pain. Patients who required removal report moderate back and leg pain until removal. When follow-up occurred after removal, patients reported mild back and leg pain. The largest difference is seen at the 24-month follow-up; patients who needed removal reported more pain than those patients who did not require removal (difference score of 3 points for back pain and 4 points for leg pain).

Patients who retained their instrumentation were also older on average, had a lower incidence of polytrauma at admission, shorter average hospital stay, and fewer levels operated on. Comorbidities did not appear to influence removal though the low rate of reported comorbidities in the current sample (15.62% of the sample) could be responsible for this finding. None of these patients required removal.

Limitations of the presented study include the fact that it is a case series so no cause and effect conclusions can be drawn. The current case study had a variable final follow-up time with the majority having final follow-up at 4 years (range 4–8). It would be beneficial to extend the follow-up to ensure facet arthrosis and possible instrument failure do not occur at a later time. However, sustaining follow-up rates becomes more problematic as the time extends. The current study has a reasonably small sample and the proportion of patients needing removal could change with a larger population. Despite the limitations mentioned given the patient outcomes reported, we would suggest that compulsory instrumentation removal is not always required following minimally invasive percutaneous screw-rod stabilization for thoracolumbar pathology. Instrumentation removal can be safely guided by clinical complaints. Average follow-up of 4 years would suggest that no risk is posed to these patients by instrumentation retention. In fact, minimal pain and disability with good work capacity can be realized despite instrumentation retention. Thus, the elimination of a second surgery for instrumentation removal as a treatment standard could be considered.

## 6. Conclusions

Minimally invasive percutaneous screw-rod stabilization for thoracolumbar pathology resulted in high levels of patient satisfaction, minimal disability, mild pain, and high return to work with low perioperative risk. These findings were sustained despite eliminating the second surgery for instrumentation removal usually performed following this nonfusion procedure. While instrumentation removal may be provided for specific indications, removal in all cases may be unnecessary.

## Figures and Tables

**Table 1:** Demographics.

	Sample (*N* = 32)		NRG (*n* = 26)		IRG (*n* = 6)	
	Mean	Range	Mean	Range	Mean	Range
Age	38.3	(18–61)	40.7	(18–61)	27.6	(19–36)
Gender						
Male	24		19		5	
Females	8		7		1	
BMI	22.1	(17.3–34.3)	23.7	(18.8–34.4)	20.8	(17.3–29.5)
Injury Severity Score	9.5	(8–41)	9	(8–34)	9.5	(9–27)
Numeric pain rating scale	7	(3–10)	7	(3–10)	7	(5–9)

	%		%		%	
Comorbidities						
Diabetic	6.2		7.7		0	
Smoker	6.2		7.7		0	
Morbidly obese	3.1		3.8		0	

Mechanism of injury						
MVC	75		73		67	
Fall	25		27		33	
Polytrauma	40.62		34.61		66.67	

**Table 2:** Diagnosis details.

Patient	Injury	AO classification	Instrumentation
1	L2 burst fracture	L2:A4, N0	Medtronic Sextant II
2	L1 chance fracture	L1:B1, N0	Medtronic Sextant II
3	T12-L1 chance fracture, L1 burst fracture	T12-L1:B2 (L1:A4), N2	Medtronic Longitude CD Horizon
4^*∗*^	T12-L1 chance fracture, L1 burst fracture, T11 compression	T12-L1:B2 (L1:A4, T11:A1), N2	Medtronic Sextant II
5	T12 burst fracture, scoliosis	T12:A4 (M2), N0	Medtronic Longitude
6	T12-L1 chance fracture	T12-L1:C, N0	Medtronic Longitude
7	T12 burst fracture, L1 burst fracture	T12:A4, L1:A4, N1	Medtronic Longitude
8	L1 burst fracture and burst fracture	L1:B1 (L1:A4), N0	Medtronic Longitude
9^*∗*^	L2 burst fracture	L2:A4, N3	Medtronic Longitude
10	T10-11 instability, T6 extension	T10-11:c, T6:B3, N0	Medtronic Legacy
11	L1 burst fracture	L1:A4, N3	Medtronic Sextant
12	T3-4 chance fracture, T4 burst fracture	T3-4:B2 (T4:A4), N3	Medtronic Longitude CD Horizon
13	T10 extension	T10:B3 (M2), N0	Medtronic Longitude
14	T11-12 chance Fracture, T12 burst fracture	T11-12:B2 (T12:A4), N0	Medtronic Sextant II
15	L1 burst fracture	L1:A4, N0	Medtronic Longitude
16	T3 chance fracture, T4 compression	T3:B1 (T3:A4), T4:A1, N0	Medtronic CD Horizon Legacy
17	T11-12 PLC, T12 burst fracture	T11-12:B2 (T12:A4), N0	Medtronic Sextant
18	T2 and T3 chance fracture, T4 compression	T2:B1, T3:B1, T4:A1, N0	Medtronic Sextant
19^*∗*^	T12 chance fracture	T12:B1, N0	Medtronic Horizon Sextant
20^*∗*^	T10 chance fracture	T10:B2, N0	Medtronic Sextant
21	L4 burst fracture	L4:A4, N0	Medtronic Sextant
22^*∗*^	T4-5 PLC, T5 chance fracture, T6 burst fracture	T4-5:c (T4:A4), T6:A4, N0	Medtronic Sextant
23	L1 burst fracture, chance fracture	L1:B1 (L1:A4), N0	Medtronic Sextant
24^*∗*^	T12 burst fracture, L1 chance fracture	L1:B1, T12:A4, N0	Medtronic Longitude
25	T10-11 PLC, T8, 11, 12 compression	T10-11:c (T11:A1), T8:A1, T12:A1, N0	Medtronic Sextant II
26	T6-7 instability	T6-7:c (T6:A4, T7:A4), N0	Medtronic Sextant
27	L1 burst fracture and PLC	T12-L1:B2 (L1:A4), N0	Medtronic Longitude CD Horizon
28	T12 burst fracture	T12:A4, N0	Medtronic Horizon Sextant
29	L5 burst fracture	L5:A4, N0	Medtronic Sextant II
30	L1 burst fracture	L1:A4, N0	Medtronic Longitude
31	T12 burst fracture and PLC	T12-L1:B2 (T12:A2), N0	Medtronic Longitude
32	L1 burst fracture, L2 compression	L1:A4, L2:A1, N0	Medtronic Sextant II

^*∗*^Patients who later require removal. PLC = posterior ligamentous complex injuries.

**Table 3:** Operative details.

OR time^*∗*^	Sample (*N* = 32)		NRG (*n* = 26)		IRG (*n* = 6)	
	Mean	Range	Mean	Range	Mean	Range
	3 h 00 min	(38 min–7 h 15 m)	2 h 51 min	(38 m–7 h 15 m)	2 h 8 min	(50 m–4 h 50 m)
Blood loss (ml)	179.6	(50–600)	188.5	(50–600)	141.6	(50–250)
Length of hospital stay (days) (median)	5	1–37	4.5	1–37	6.5	1–15
Number of levels (median)	2	1–6	2	1–6	3.5	2–5

^*∗*^OR time was inclusive of preparation time, induction, incision, closure, and patient being transferred onto the floor.

**Table 4:** Pain and disability scores.

	NRG			IRG		
	NRS-B	NRS-L	ODI	NRS-B	NRS-L	ODI
3 months	4	4.5	26	6	5	23
12 months	3	2	23	4	4	21
24 months^*∗*^	2	1	21	5	5	27
48 months^*∗∗*^	2	1	16	3	3	14

^*∗*^24-month follow-up ranged for the IRG cohort from 16 to 24 months depending on time of instrumentation removal. ^*∗∗*^This is an average of final follow-up periods which ranged from 4 to 8 years depending on time of surgery.

## Data Availability

The data used to support the findings of this study were from the patient's medical records and the Canadian Spine Society Registry Study forms. Medical records were supplied by Horizon Health Network and so cannot be made freely available. The Research Ethics Board for the Horizon Health Network is organized and operates according to the principles of the ICH Harmonized Tripartite Guidelines: Good Clinical Practice, Tri-Council Policy Statement, and Division of the Food and Drug Regulations. Requests for access to deidentified data from the study forms should be made to Dana El-Mughayyar at dana.el-mughayyar@horizonnb.ca.
